# Adverse childhood experiences contribute to blood pressure changes in adulthood: a meta-analysis of over 750 000 adults

**DOI:** 10.7189/jogh.15.04100

**Published:** 2025-06-13

**Authors:** Wenjing Li, Liping Pu, Yingtao Meng, Xiaoning Wang, Liang Dong, Yirong Yang, Hao Liu, Xinai Wang, Jingying Liu, Chenqi Wang, Yaxuan Wu, Hongling Xiao

**Affiliations:** 1 Cancer Hospital of Shandong First Medical University, Shandong, China; 2 Suzhou Vocational Health College, Jiangsu, China; 3 Ruijin Hospital, Shanghai Jiaotong University School of Medicine, Shanghai, China; 4 Ruijin-Hainan Hospital, Shanghai Jiao Tong University School of Medicine, Hainan, China; 5 Nursing Department of Tianjin People's Hospital, Tianjin, China; 6 Tianjin Medical University Cancer Hospital, Tianjin, China; 7 Tianjin University of Traditional Chinese Medicine, Tianjin, China; 8 Zhejiang Chinese Medical University, Zhejiang, China

## Abstract

**Background:**

Adverse childhood experiences (ACE) have been linked to various negative health outcomes in adulthood. However, their relationship with blood pressure remains inconclusive. We aimed to conduct a systematic review and meta-analysis to investigate this association.

**Methods:**

We conducted a comprehensive search of PubMed, Cochrane Library, Scopus, Web of Science, China National Knowledge Infrastructure, Wanfang Data, and VIP from the databases' inception until 1 July 2023. We assessed the quality of included cross-sectional and cohort studies using the Agency of Healthcare Research and Quality and Newcastle-Ottawa Scale criteria. We analysed the combined effect size for the diagnosis of hypertension and changes in systolic blood pressure and diastolic blood pressure from the included studies further explored these results based on geographical and age subgroups.

**Results:**

We included a total of 19 cohort and 23 cross-sectional studies, comprising 754 146 participants and investigating 24 types of ACE. In addition to parental divorce or separation and group violence, exposure to domestic violence, physical neglect, and physical abuse were associated with an increased risk of hypertension, with the risk increasing with the number of exposures. This association was prominent in Europe and North America and in adults. However, the relationship varied among Asian populations and minors due to the type and degree of ACE.

**Conclusions:**

Our results provide evidence supporting the association between ACE and elevated blood pressure, suggesting that ACE may contribute to the development of hypertension in adulthood. Future studies should explore potential gender differences in this association.

**Registration:**

PROSPERO: CRD42023442287

Adverse childhood experiences (ACE) consist of one or more negative events of psychological or physical harm and threat before the age of 18, including issues such as childhood abuse, family dysfunction, and collective violence [1]. The global prevalence of ACE is high, affecting over 60% of minors, with approximately 20% enduring four or more ACEs concurrently [2]. These traumatic occurrences not only significantly impact children's mental health but also pose potential threats to their physical health as adults [3].

Recent research has focussed on the association between ACE and alterations in blood pressure (BP) during adulthood, aiming to explain the underlying mechanisms and provide a scientific foundation for early prevention and intervention measures [4]. Abnormal BP changes are a crucial indicator for assessing cardiovascular health, often predictive of an increased risk of cardiovascular disease. Notably, hypertension is a leading cause of cardiovascular morbidity and mortality [5], and its regulation is influenced by various factors, including genetics, environment, and lifestyle. Among these, there is mounting evidence that ACE constitute a significant risk factor for the development of hypertension [6,7]. These adverse experiences, such as domestic violence, sexual abuse, and emotional neglect, can leave children under chronic stress for prolonged periods. This persistent state of stress activates the body's stress response systems, including the sympathetic nervous system and the hypothalamic-pituitary-adrenal axis [8], ultimately leading to an increase in BP [9–11].

However, current research investigating the relationship between ACE and alterations in BP has yielded inconsistent findings. Some studies have established a significant correlation between ACE and elevated BP in adulthood, while others have not detected such an association [4,12]. A meta-analysis showed that angiotensin-converting enzyme was linked to BP in 61.5% of the studies reviewed [13]. This disparity in results may stem from a range of variables, including gender [4], age [14], and the specific nature of ACE encountered [15]. In certain instances, distinct types of ACE have been associated with a reduction in BP.

To gain a more precise understanding of this relationship, we conducted a systematic review and meta-analysis, aiming to derive more definitive conclusions and provide a scientific rationale for a deeper comprehension of the link between ACE and hypertension, thereby facilitating subsequent preventive and interventional measures.

## METHODS

We registered the study on PROSPERO (CRD42023442287) and reported our findings following the PRISMA guidelines [16].

### Search strategy

Based on the World Health Organization (WHO) definition of ACE and related research [1], we formulated a comprehensive search using terms and keywords ‘blood pressure’, ‘hypertension’, ‘adverse childhood experiences’, and those related to various types of ACE (Tables S1 and S2 in the **Online Supplementary Document**). We searched PubMed, Cochrane Library, Scopus, Web of Science, China National Knowledge Infrastructure, Wanfang Data, and VIP, from the inception of each database until 1 July 2023. We did not apply language restrictions to this search. Two researchers (CW, YW) who had undergone uniform training used NoteExpress, version 4.X (Aegean Software Co, Ltd., Beijing, China) to review the titles and abstracts of the retrieved records to determine their eligibility. Following this stage, they read the records meeting the requirements in full and included those meeting the inclusion criteria. In cases of disagreement between the two reviewers, a third independent reviewer was consulted.

### Inclusion and exclusion criteria

We included studies that examined one or more ACEs and compared individuals with ACEs to control individuals with no or fewer ACEs, with outcome indicators including the diagnosis of hypertension or BP measurements. We excluded studies with incomplete data extraction from experiments, duplicate studies from the same database, or those involving randomised controlled trial, quasi-experimental studies, single-group studies, study protocols, systematic reviews, syntheses, conference proceedings, or animal experiments.

### Data extraction

After screening the titles, abstracts, and full texts of the studies, two researchers who had undergone uniform training independently conducted data extraction, cross-checking it afterwards and consulting a third researcher for resolution of any disagreements. This included data on the authors (*i.e.* author name and surname, year), the study population (*i.e.* study type, country, sample size, age, and gender), ACE (*i.e.* definition, number, type, measurement, reporting method, and reporting time), and BP (*i.e.* reporting method, reporting time, measurement, and the amount of effect presented in various forms). We contacted authors by email if study results (*i.e.* hypertension and BP indicators) were incomplete and excluded studies if they failed to provide the required data.

### Quality assessment

Two researchers independently performed the risk of bias evaluation of the included literature and cross-checked the results, resolving discrepancies with a third researcher. We evaluated the cross-sectional studies using the criteria recommended by the Agency of Healthcare Research and Quality [17], which consisted of a total of 11 entries, with a score of 0–3 indicating low quality, 4–7 indicating moderate quality, and 8–11 high quality. We evaluated the quality of the included case-control and cohort studies using the Newcastle-Ottawa Scale [18], which consisted of a total of nine entries, with a score of 0–3 indicating low quality, 4–6 indicating moderate quality, and 7–9 indicating high quality. To ensure the quality of the literature, we only included those scored as medium to high quality.

### Statistical analyses

We conducted the meta-analyses using Stata, version 17.0 (StataCorp, College Station, Texas, USA) and R, version 4.2.2 (R Core Team, Vienna, Austria). We used the odds ratio (OR) to pool the data. If articles reported relative risk (RR) or hazard ratio (HR), we transformed them using the formula *RR* = *OR* / (1 − *p0* + (*p0* × *OR*)), where p_0_ represents no ACE exposure [19]. We assessed heterogeneity using Cochran’s Q test and the *I^2^* heterogeneity test and performed subgroup or sensitivity analyses for data with *I^2^*>50% heterogeneity. When heterogeneity between studies could not be eliminated using the above methods, we employed a random-effects model for the analyses. Additionally, we conducted sensitivity analyses on indicators from three or more studies to assess the impact of individual studies on the pooled results and used subgroup analyses to explore potential sources of heterogeneity. When more than seven studies were used to pool the data, we quantified it by Egger's test (test level α = 0.05) to investigate whether it had publication bias.

## RESULTS

**Figure 1:**
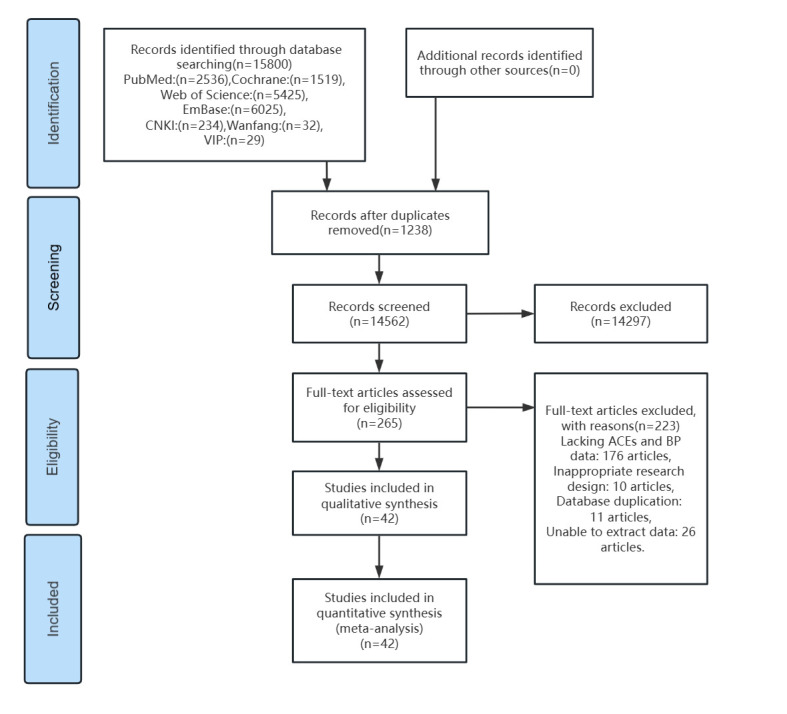
Flowchart of the review process.

The search of the seven databases yielded a total of 15 800 records. After removing duplicates, records dealing with irrelevant topics or with inappropriate study designs, those lacking outcome measures, and those with <100 participants, we were left with 42 studies, of which 40 were in English and two in Chinese (**Figure 1**). These studies reported on 24 categories of ACE (**Table 1**), as well as 35 hypertension and 11 BP indicators.

**Table 1:** Types of ACE included in the study

Type of ACE	n
Abuse and neglect	6
Neglect	13
Emotional neglect	9
Physical neglect	9
Abuse	2
Emotional abuse	19
Physical abuse	23
Sexual abuse	23
Family dysfunction	4
Parental divorce or separation	13
Parental deaths	2
Domestic violence	8
Household mental illness	8
Household substance abuse	8
Household crime	9
Peer bullying	3
Community violence	3
Group violence	4
One or more types of ACE	7
One or mild ACE	12
Two or moderate ACE	10
Three ACE	12
Four or severe ACE	9
Accumulated per ACE	4

ACE – adverse childhood experience

### General characteristics of participants

The 42 included records (Table S1 in the **Online Supplementary Document**) included 23 cross-sectional and 19 cohort studies from North America (n = 21), Europe (n = 11), Asia (n = 8), South America (n = 2), and Africa (n = 2). There were 754 146 participants, of whom 742 394 had data on their gender, with 51.63% being female.

Among the definitions of ACE, seven studies used the Child Trauma Questionnaire [20], four used the Childhood Family Environments Questionnaire [21], and three used the Adverse Childhood Experiences International Questionnaire [22]. Most studies (n = 28) used self-reports of ACE, five used face-to-face interviews, and five used institutional or government records. Sexual (n = 27), physical (n = 27), and emotional (n = 21) abuse were the most reported ACEs. Regarding the measurement of BP, approximately half of the articles (n = 23) did not explicitly report the criteria employed. However, most of the studies (n = 30) relied on professional assessments for measuring BP, ensuring a standardised and reliable approach to BP measurement across the included literature.

### Quality of articles and risk of bias

We assessed 23 cross-sectional studies using the Agency of Healthcare Research and Quality criteria, of which 18 were of high quality and five were of moderate quality (Table S3 in the **Online Supplementary Document**). The risk of bias was mainly due to missing response rates (n = 20) and single BP measurements (n = 17). We used the Newcastle-Ottawa Scale assessment in 19 cohort studies, of which 11 were of high quality and eight of moderate quality (Table S4 in the **Online Supplementary Document**). The lower ratings were mainly the result of failure to exclude patients with baseline hypertension (n = 11), failure to mention the dropout rate or dropout >20% without describing the characteristics of those lost to follow-up (n = 13), or failure to collect population-based data (n = 7).

When testing for publication bias, the results of the Egger test, which categorises ACE based on the fact that the studies in question do not analyse the confounding effect of the full range of ACE categories, showed no significant publication bias (Table S5 in the **Online Supplementary Document**).

### Meta-analysis of exposure to ACE

In the analysis without gender stratification, exposure to a single type of ACE ranged from 33.7% (group violence) to 9.5% (sexual abuse), and combined exposure ranged from 35.8% (one or more types of ACE) to 10.7% (three types of ACE) (**Table 2**). Subgroup analyses revealed significant differences in exposure by gender. Males were more exposed to emotional neglect, physical neglect, domestic violence, community violence, and group violence. Females were more likely to be sexually abused and to experience cumulative adversity in terms of multiple abusive neglect and family dysfunction.

**Table 2:** Exposure to ACE*

Type of ACE	Total	Male	Female
Abuse and neglect	25.2	28.2	44.6
Neglect	17.7	7.9	14.9
Emotional neglect	21.2	41.1	30.9
Physical neglect	21.1	32.0	22.3
Abuse			33.7
Emotional abuse	27.1	22.5	25.0
Physical abuse	17.7	14.9	17.7
Sexual abuse	9.5	11.2	15.6
Family dysfunction	23.3	23.1	37.4
Parental divorce or separation	19.8	17.8	20.0
Parental death	10.0		
Domestic violence	22.7	46.1	28.7
Household mental illness	13.6	7.7	10.5
Household substance abuse	25.6	36.7	32.4
Household crime	9.5	8.5	6.0
Peer bullying	31.6	25.3	14.7
Community violence	24.1	29.0	18.7
Group violence	33.7	41.1	28.3
One or more types of ACE	35.8		28.2
One or mild ACE	25.4	31.9	33.8
Two or moderate ACE	14.7	19.3	19.4
Three ACE	10.7	19.9	12.4
Four or severe ACE	22.5	20.8	19.5

ACE – adverse childhood experience

*Values are expressed as percentages.

### Meta-analysis of type and number of ACE and BP

We analysed the 35 studies in which the outcome indicator was hypertension (Table S2 in the **Online Supplementary Document**). There were more studies on sexual abuse and physical abuse (>10), and pooling the results for each type of ACE showed that domestic violence, physical neglect, and physical abuse contributed more to hypertension among individual types of ACE. Four or severe ACE, three ACE, abuse, and neglect as multiple different types of overlapping exposures were also associated with a diagnosis of hypertension in adulthood, and each additional exposure to ACE significantly increased the odds of an individual developing hypertension (adjusted OR (aOR) = 1.06; 95% confidence interval (CI) = 1.05, 1.07).

We performed the subgroup analyses for different countries (Table S2 in the **Online Supplementary Document**). Research from Asia indicates that there are associations between physical neglect, emotional abuse, and two, four or severe exposures and hypertension. On the other hand, the evidence from Europe and North America all supported the association between ACE and hypertension.

However, subgroup analyses by age showed that data for minors did not strongly support this association, except for four or severe cumulative exposures (aOR = 1.34; 95% CI = 1.20, 1.49) (Tables S2 and S3 in the **Online Supplementary Document**). When the age range was restricted to young and middle-aged adults, we observed an increase in the types of ACE associated with BP. When this age range was extended to full adulthood, the associations between abuse and neglect, emotional abuse, sexual abuse and hypertension were attenuated.

We also considered the degree of fluctuation in systolic blood pressure (SBP) and diastolic blood pressure (DBP) in our analysis (**Table 3**). Only three studies provided data on parental separation or divorce; here, we found that that neither SBP nor DBP was affected. The remaining types of ACE were covered by only 1–2 studies, showing that individual studies support neglect, household crime, one or more types of ACE, and four or more severe ACE increase SBP or DBP, and suggesting that group violence decreases DBP.

**Table 3:** Effects of different types and amounts of ACE on BP

	SBP		DBP	
**Type of ACE**	**n**	**ES (95% CI)**	**n**	**ES (95% CI)**
Abuse and neglect	2	2.31 (−3.20, 7.82)	2	−0.80 (−4.92, 3.31)
Neglect	1	1.40 (0.51, 2.29)	1	0.99 (0.39, 1.60)
Emotional abuse	1	0.03 (−0.09, 0.15)	2	0.15 (−0.20, 0.50)
Physical abuse	2	0.04 (−0.10, 0.19)	2	0.14 (−0.02, 0.30)
Sexual abuse	2	−1.14 (−3.89, 1.61)	2	−0.80 (−2.73, 1.12)
Parental divorce or separation	3	2.16 (−0.76, 5.07)	3	0.59 (−1.07, 2.24)
Household crime	1	6.64 (0.89, 12.40)	1	4.14 (−0.02, 8.30)
Group violence	1	−0.10 (−0.21, 0.01)	1	−0.80 (−0.86, −0.74)
One or more types of ACE	1	0.73 (0.50, 0.97)	1	0.83 (0.64, 1.02)
One or mild ACE	1	−0.10 (−0.54, 0.34)	1	0.18 (−0.12, 0.48)
Four or severe ACE	1	1 (0.60, 1.41)	1	0.2 (−0.20, 0.60)
Accumulated per ACE	1	−0.10 (−0.63, 0.43)	1	0.18 (−0.18, 0.54)

ACE – adverse childhood experience, BP – blood pressure, CI – confidence interval, DBP – diastolic blood pressure, ES – effect size, SBP – systolic blood pressure

### Sensitivity analyses

We also analysed sensitivity by excluding studies on a case-by-case basis. When we could not extract non-sex-stratified data for the outcomes measures from the original studies, male and female subgroup data were separately included in the analysis. Finally, from one article [23], we only excluded data related to one dimension (emotional abuse). The sensitivity results for the other categories showed no meaningful changes from the primary outcome.

## DISCUSSION

Our results suggest that there exists a correlation between ACE and an increased risk of hypertension development in adulthood, which is further modulated by age and national geographic context. We observed a progressive increase in hypertension risk with each additional exposure to ACE, indicating that a greater number of ACEs experienced translates to a higher risk of hypertension development. This underscores the cumulative impact of ACEs on long-term health outcomes.

### Mechanisms and differences in types of ACEs causing hypertension

The current understanding of the underlying mechanisms of ACE in causing hypertension revolves primarily around three interconnected domains: physiological pathways, mental health, and subsequent adverse behavioural patterns [8].

Initially, exposure to ACEs prompts irregular activation of the hypothalamic-pituitary-adrenocortical axis and the sympathetic-adrenomedullary system [24]. This activation disrupts the normal functioning of the autonomic nervous system, resulting in elevated resting BP and heart rate [25], as well as potentially deleterious changes in cardiovascular parameters, such as cardiac output and peripheral resistance [26]. Mental health issues occupy a crucial position in this interrelation [27]. These experiences are known to trigger mental health problems [28], including anxiety and depression, which in turn heighten the risk of cardiometabolic morbidity and mortality [29]. Lastly, adverse health behaviours often bridge the gap between ACE and hypertension. For instance, individuals who have endured multiple adversities may be predisposed to resorting to addictive substances and substance abuse as coping mechanisms due to immature brain development [30]. This poor health coping mechanism may trigger health problems such as obesity and overweight in adulthood as one of the delayed consequences of ACE leading to increased BP [31].

### Divergences in the types of ACEs leading to hypertension

Abuse and neglect cover the main five subtypes of ACEs and have a strong association with hypertension. Physical abuse may lead children to adopt hostile attitudes towards relationships due to emotional neglect [32]. Individuals who have experienced sexual abuse could face extended victimisation, harm to their reputation, and loss of chastity, potentially leading to symptoms of mental health problems and somatisation [33]. Damage to the developing brain caused by emotional abuse can adversely affect emotional and social-cognitive development [34]. Emotional neglect is also significantly associated with psychological maladjustment and negative personality traits. Furthermore, people who are subjected to stressful situations often turn to substandard coping mechanisms, such as smoking, drinking, emotional overeating, reduced physical activity [35,36], and lack of sleep [29]. These actions have the potential to cause harmful changes in BP. Physical neglect during a period of rapid physical development increases the preference for high-fat foods and changes the metabolism and composition of the adrenal axis and adipose tissue, which creates a more favourable environment for energy storage [37].

Family dysfunction can also contribute to hypertension. The passing of a parent diminishes health maintenance resources, including economic and psychological support [38], resulting in slower BP response and recovery [25]. Additionally, hereditary susceptibility to illness from early parental death also adds to an individual's BP [39]. Different from the permanent separation of parents, we did not find the connection between parents' divorce and BP in our research. The impact that separation has on children's health is influenced by the presence or absence of intense conflict before this event occurs [40]. Family function, rather than marital structure, is a more reliable predictor of health outcomes for minors [41], which is highly stressful for children. We additionally discovered that hypertension risk is increased by family violence, the existence of drug-abusing, incarcerated, or mentally ill family members, as well as familial genetic and behavioural influences.

### Cumulative effects of ACE on hypertension

ACEs frequently take on the form of cumulative multipliers that result in individuals being subject to patterns of persistent or multiple victimisation [42]. Children who have encountered sexual abuse are at least two times more likely to experience neglect, maltreatment, domestic violence, and parental substance misuse again when compared to children who have not experienced ACE [35]. The more ACEs an individual reports, the more likely they are to suffer from emotional and somatic morbidity and develop health-harming behaviours. Additionally, a higher number of exposures correlates with a statistically significant increase in the risk of heart disease and depression [43]. Studies in both developed [44] and developing countries [45] have demonstrated this cumulative effect. In our analysis, we discovered that a higher number of exposures to ACE, as opposed to the type of ACE, had a greater effect on the increase in BP. Furthermore, the cumulative number of exposures was found to increase the risk of hypertension. Exposure to multiple types of neglect, abuse, and family dysfunction resulted in a greater risk of developing the condition. The risk of hypertension continued to remain elevated with increasing types of ACE, even without disaggregating them.

### Demographic differences of ACE causing hypertension

Age is a significant demographic factor influencing BP. However, upon analysing data from minors, we observed no substantial correlation with BP. This lack of correlation primarily stems from the fact that the potential adverse effects of ACE on downstream BP manifest gradually over an extended period [8,46]. During early stages, these effects are often subtle and challenging to recognise directly. Our findings for the young and middle-aged cohorts aligned with the overall analysis, revealing a certain correlation between ACE and BP. Nevertheless, this correlation seems to diminish among older age groups. This may be partly due to the natural increase in BP with age, potentially masking the impact of ACE on BP. Furthermore, potential biases in older individuals' recollections of past experiences [2,47], coupled with the premature mortality linked to ACE [48], could affect both the accuracy of the ACE questionnaire responses and the representativeness of the sample, especially among those aged >65 years.

Geographical disparities introduce another layer of complexity to this relationship. Distinct cultural backgrounds and social practices across various regions shape an individual's upbringing and stress levels. For instance, parents in Asian cultures often assume full responsibility for their children's upbringing [49], sometimes imposing excessive pressure that might be deemed abusive in Western contexts. Studies indicate that psychological identity differences among Asians due to cultural variations could contribute to divergent BP responses compared to other populations [50]. Psychological identity, referring to an individual's beliefs and perceptions about experiencing adverse events, significantly impacts both somatic and psychological reactions. Different cultural backgrounds can influence how people interpret and respond to ACE [50], thereby affecting the mechanism by which ACE impact health indicators like BP.

Besides age and geography, gender and family history also play a pivotal role in hypertension development. Females, influenced by socio-cultural norms, tend to be more susceptible to violence, potentially leading to greater health deterioration due to ACE [51]. Hypertension also exhibits significant familial clustering, with individuals having a family history of hypertension at an elevated risk of developing the condition [52,53]. Additionally, unhealthy lifestyle choices, such as diets rich in salt, sugar, and fat, persistent mental stress, being overweight or obese, smoking, and excessive alcohol consumption, are all established hypertension risk factors [53]. Nevertheless, limitations in the available raw data prevented an in-depth exploration of these factors in our analysis.

### Strengths and limitations

There are several strengths of our study. First, we acknowledge the pivotal role of age in investigating the ACE-BP relationship and have broadened the geographical scope of our sample, enabling a more comprehensive examination of this association. This approach allowed us a deeper understanding of the ACE-BP outcomes. Furthermore, our analyses encompassed not only hypertension diagnosis but also the intricate trends of SBP and DBP, offering a comprehensive evaluation of ACE's impact on BP.

However, we must also acknowledge some limitations. While we recognise the potential influence of gender in the ACE-BP relationship, the limited data availability within each gender subgroup prevented an in-depth integrated analysis. Additionally, the BP data relied solely on a single measurement and self-reported hypertension diagnosis, potentially introducing bias. Finally, the cross-sectional design of the study somewhat constrained our ability to delve deeper into the causal relationship between ACE, BP fluctuations, and hypertension diagnosis.

Another limitation lies in the inconsistent measurement criteria employed across the included articles. The use of different scales to explore ACE exposure, particularly in retrospective adult studies, introduces potential challenges to the accuracy and reliability of the results. Subjects' recollection of their early traumatic experiences may be influenced by factors such as forgetting, distorting, repressing, or identifying with these events, thereby impacting the validity of the findings. Additionally, relying solely on official administrative records, rather than self-reports, may overlook minor cases that have not yet manifested serious consequences. Furthermore, the reliance on self-reported hypertension diagnoses often leads to patient neglect, resulting in lower prevalence rates being captured. These limitations could contribute to an underestimation of ACE exposure and a subsequent underdiagnosis of hypertension, potentially influencing our results.

We also note issues in the inadequate control of covariates and mediating variables. Numerous factors have been identified as influential in the development of hypertension, and without effectively controlling covariates such as family history, establishing a link between ACE and the disease becomes challenging, particularly in cross-sectional studies. Moreover, maladaptive behaviours and cascading pathogenetic mechanisms related to emotional cognition mediate the relationship between ACE and BP. Some studies have demonstrated that the correlation between ACE and adult hypertension weakens when considering additional factors such as psychiatric disorders (*e.g.* depression) and behaviours like smoking, alcohol consumption, and diet. The inconsistent control or inclusion of these mediating variables within the analysed studies limited us from accurately determining the proportionate contribution of ACE to BP outcomes.

### Directions for future research

First, establishing standardised measurement systems and implementing multimodal data validation will be critical. Harmonised ACE assessment tools cross-culturally validated through refinements to the regional applicability of tools like the ACE-IQ questionnaire could mitigate biases from inconsistent scales. Integrating dynamic physiological monitoring techniques such as continuous BP monitoring and HR variability assessments, alongside biomarker profiling including diurnal cortisol patterns and inflammatory markers like interleukin-6, would complement self-reported data to enhance methodological rigour. To address recall bias, combining the life history calendar method with contextually salient events like family relocations or educational transitions could improve the spatiotemporal precision of retrospective trauma reporting.

Second, elucidating dynamic mediation mechanisms necessitates integrating longitudinal designs and advanced statistical methods. Given the temporal variability and individual differences in ACE-related pathways influencing blood pressure, longitudinal mediation models combined with machine learning-driven analyses could decode these complexities. For example, reinforcement learning may pinpoint critical periods for mediators such as depressive symptoms or smoking, while metabolomics could delineate neuroendocrine contributions across developmental stages. Causal inference frameworks should disentangle genetic risks and gene-environment interplay, particularly in multigenerational cohorts, clarifying direct ACE effects versus intergenerational transmission pathways.

## CONCLUSIONS

We identified 24 ACEs demonstrating significant associations with hypertension, with key findings being that hypertension shows differential correlation intensities with individual ACEs subtypes, and that cumulative ACEs exposure exhibits a compound effect, where multi-type adversities synergistically amplify hypertension risk through dose-response relationships. Studies conducted in European and North American populations yielded more pronounced associations compared to Asian cohorts, potentially attributable to cultural context variations. The pathophysiological impact of ACE manifested progressively, with BP alterations becoming most pronounced during early to mid-adulthood. Future investigations should prioritize examining gender-specific variations in these exposure-outcome relationships.

## Additional material


Online Supplementary Document
