# Supplementary material for: Adverse childhood experiences contribute to blood pressure changes in adulthood: a meta-analysis of over 750 000 adults
Source: J Glob Health. 2025 Jun 13;15:04100. doi: 10.7189/jogh.15.04100 (PMC12161486; doi:10.7189/jogh.15.04100)
Supplement: Online Supplementary Document [file jogh-15-04100-s001.pdf]

Supplement to: Li W, Meng Y, Pu L, Wang X, Dong L, Yang Y, Liu H, Wang X, Liu J, Xiao H, Wang C, Wu Y. Adverse childhood experiences contribute to blood pressure changes in adulthood: a meta-analysis of over 750 000 adults. J Glob Health. 2025;15:04100.

Table S1. Basic data for inclusion of articles

First Author	Year	Country	Study Type	Databases used	Sample Size	Sample Type	Percent Female	Data access time			ACE				BP		Evaluation criteria	Score
								Age	ACE	BP	Definitions	Number of collections	Types of analysis	Report	Criteria	Report		
Aguayo	2022	US	C	CARDIA	3642	18-40 years	56.00 %	NA	15 years after baseline	30 years after baseline	CFEQ	3	⑳,㉑	Self-reported	140/90	Trained personnel	NOS	8
Alastalo	2009	Finnish	C	NA	2003	Adults	53.67 %	NA	1939	2001-2004	Self-encoding	1	⑩	Government records	NR	Trained personnel	NOS	7
Alhowaymel	2022	Saudi Arabia	CS	NA	338	Rural Resident Adults	37.00 %	26.3 ±8.9	NA	NA	ACE Questionnaire	10	②,⑥,⑦,⑧,⑨,⑳,㉑,㉒,㉓	Self-reported	NR	Physician-confirmed Self-report	AHRQ	8
Almuneef	2017	Saudi Arabia	CS	NA	10156	Adults	47.76 %	34.3 ± 11.3	NA	NA	ACE-IQ	13	②,⑥,⑦,⑧,⑩,⑫,⑬,⑭,⑮,⑯,⑰,⑱,⑳,㉑,㉒,㉓	Self-reported + face-to-face interviews	NR	Physician-confirmed Self-report	AHRQ	8
Andrade	2021	US	CS	NA	133	Hispanic youth ages 15-21	60.20 %	19.1	NA	NA	CFEQ + Self-encoding	11	③,④,⑥,⑦,⑧,⑩,⑫,⑬,⑭,⑮,⑯,㉓	Self-reported	NR	Trained personnel	AHRQ	9
Carson	2022	US	C	MsHeart	162	Late perimenopausal and postmenopausal women	100.00 %	NA	40 to 60 years	ACE measured 5 years later	CTQ	5	①,③,④,⑥,⑦,⑧	Self-reported	NR	Trained personnel	NOS	8
Chandan	2020	UK	C	THIN	241971	Population-based	41.68 %	NA	18 years and older	One year after baseline or death	Official confirmation	13	⑲	Official records	NR	Doctor's report	NOS	5
Chen	2021	Sweden	CS	NA	48624	Males 18-20	0.00%	18-20	NA	NA	Official confirmation	4	⑪,⑳,㉑,㉒	Government records	140/90	Trained personnel	AHRQ	7
Clemens	2021	German	CS	A representative	2510	>14 years	53.30 %	48.4 ±18.2	NA	NA	CTQ	5	①,②,③,④,⑤,⑥,⑦,⑧	Self-reported	NR	NR	AHRQ	9

				sample of the German population														
Dorji	2020	Bhutan	CS	NA	337	>60 years	43.90%	60-101	NA	NA	ACE-IQ after adjustment	13	④,⑥,⑦,⑩,⑫,⑬,⑭,⑮,⑯,⑰,㉒,㉓	Face-to-face interviews	NR	Self-reported	AHRQ	8

Continued

First Author	Year	Country	Study Type	Databases used	Sample Size (N)	Sample Type	Percent Female	Data access time			ACE				BP		Evaluation criteria	Score
								Age (years)	ACE	BP	Definitions	Number of collections (n)	Types of analysis	Report	Criteria (mm Hg)	Report		
Eskenazi	2019	US	C	CHAMACOS	397	Minors	52.10%	NA	16 years	14 and 16 years	A portion of PIPES	1	⑱	Self-reported	>95th percentile for age and sex	Trained personnel	NOS	4
Flores-Torres	2020	Mexico	C	MTC	9853	Female Teachers 25 years and older	100.00%	NA	2014-2016	2006-2008 + 2011-2014	CFEQ	10	②,③,④,⑤,⑥,⑦,⑧,⑨,⑩,⑫,⑬,⑭,⑮,⑲,⑳,㉒, ㉔	Self-reported	NR	Physician-confirmed Self-report	NOS	6
Ford	2014	US	C	Add Health	7971	Minors	55.40%	NA	11-17 years	23-30 years	Self-encoding	2	⑰	Face-to-face interviews	140/90	Trained personnel	NOS	8
Goncalves Soares	2021	UK	C	ALSPAC	3223	Minors	66.50%	NA	22 years	18、25 years	CTQ-SF + Sexual Experiences Survey	3	⑥,⑦,⑧	Self-reported	NR	NR	NOS	5
Goodwin	2004	US	CS	NCS	8089	15-54 years	52.55%	15-54	NA	NA	A checklist of life events	3	②,⑦,⑧	Self-reported	NR	Self-reported	AHRQ	9
Halonen	2015	Finnish	C	FPSS	37699	Public sector employees	78.70%	NA	2008-2009	2014	SLC	6	⑨	Self-reported	NR	Doctor's report	NOS	6
Hc	2014	US	CS	Add Health	11384	Adolescents	52.96%	24-32	NA	NA	CTS①	3	⑥,⑦,⑧	self-reported	140/89	Trained personnel	AHRQ	11
Islam	2021	US	CS	MECA	457	Black or African American	62.00%	53±10	NA	NA	ETI	4	⑥,⑧,⑲	Self-reported	140/90	Self-reported	AHRQ	9

						30-70 years												
Keogh	2022	US	C	MIDUS	2568	Adults	49.34 %	NA	1995	2013-2014	Self-encoding	3	⑲	Self-reported	130/80	Physician-confirmed Self-report	NOS	8
Kim	2021	Uganda	CS	whole-population social network cohort study	545	Adults	62.00 %	45.9 ±15.9	NA	NA	ACE-IQ	16	⑥,⑦,⑧,⑩,⑬,⑭,⑮,㉔	Self-reported	140/90	Self-reported	AHRQ	9
Kreatsoulas	2019	US	CS	BRFSS	45482	Adults	59.70 %	57.9 ±15.2	NA	NA	Self-encoding	10	⑥,⑦,⑧,⑫,⑬,⑭,⑮,⑳,㉑,㉒,㉔	phone interviews	NR	Doctor's report	AHRQ	8

Continued

First Author	Year	Country	Study Type	Databases used	Sample Size (N)	Sample Type	Percent Female	Data access time			ACE				BP		Evaluation criteria	Score
								Age (years)	ACE	BP	Definitions	Number of collections (n)	Types of analysis	Report	Criteria (mm Hg)	Report		
Li	2019	UK	C	NCDS	9310	Minors	NR	NA	7、11、45 years	45 years	Self-encoding	4	②,⑥,⑦,⑧,⑳,㉑,㉒,㉔	Self、teacher、parent-report	NR	Trained personnel	NOS	8
Lin	2021	China	CS	CHARLS	11972	>45 years	51.60 %	59.85±9.56	NA	NA	Self-encoding	12	⑳,㉑,㉒,㉓	Face-to-face interviews	140/90	Physician-confirmed Self-report	AHRQ	9
Moore	2021	US	CS	NA	100	18-30 years	73.00 %	19.20±2.13	NA	NA	CTQ	5	①,③,④,⑥,⑦,⑧	Self-reported	135/85	Trained personnel	AHRQ	7
Nguyen	2022	South Africa	CS	RICE	1797	Females 18-40	100.00 %	24(21–29)	NA	NA	CTQ-SF	4	①,②,⑥,⑦,⑧,⑳,㉒,㉔	Self-reported	140/90	Trained personnel	AHRQ	10
Parrish	2013	Brazil	CS	SPMHS	5037	Adults	53.00 %	≥18	NA	NA	As defined by WMH	4	②,⑦,⑧,⑫	Self-reported	NR	Self-reported	AHRQ	9

Ramiro	2010	Philippine	CS	NA	1068	>35 years	49.90%	46.7±9.2	NA	NA	CFEQ	11	③,④,⑥,⑦,⑧,⑩,⑫,⑬,⑭,⑮,⑳,㉑,㉒,㉓	Face-to-face interview	NR	Self-reported	AHRQ	9
Rebbe	2018	US	C	Midwest Study	595	Minors	NA	NA	17 years	25-26 years	Official confirmation	3	㉓	Agency records	NR	NR	NOS	4
Reid	2018	US	CS	a cohort of postinstitutionalized youths and a nonadopted youths	120	8-18 years	50.00%	13.13	NA	NA	Official confirmation	1	⑪	Agency records	Determination of BP percentile from age, sex and height	Trained personnel	AHRQ	7
Riley	2010	US	C	NHS II	41792	Female Nurses 25-44	100.00%	NA	1995、2001	2003	CTS①	2	⑦,⑧,⑳,㉑,㉒,㉓	Self-reported + phone interviews	140/90	Doctor's report	NOS	7
Roberts	2008	US	C	NA	1110	African Americans aged 25–50 years	63.00%	NA	1988	2001	EDS	2	⑱	Self-reported	140/90	Trained personnel	NOS	8
Roettger	2022	Australia	C	MUSP	1712	Minors	NR	NA	5、14 years	21、30 years	Self-encoding	1	⑮	Mothers' report	140/90	Trained personnel	NOS	8
Ruobing Song	2019	China	CS	NA	795	18-59 years	60.20%	43.70±7.30	NA	NA	FES-CV + CTQ-SF	5	③,④,⑥,⑦,⑧	Self-reported	140/90	Trained personnel	AHRQ	10

Continued

First Author	Year	Country	Study Type	Databases used	Sample Size (N)	Sample Type	Percent Female	Data access time			ACE				BP		Evaluation criteria	Score
								Age (years)	ACE	BP	Definitions	Number of collections (n)	Types of analysis	Report	Criteria (mm Hg)	Report		
Schreier	2019	US	CS	PCS3	213	Adults	42.25%	30.1 ± 10.8	NA	NA	RFQ + FES	13	⑲	Self-reported	NR	Trained personnel	AHRQ	7
Sheikh	2018	Norway	CS	the Tromsø Study	10765	Adults	51.10%	57.5±12.6	NA	NA	Self-encoding	6	⑲	Self-reported	NR	Self-reported	AHRQ	8
Soares	2020	UK	C	the UK Biobank	157311	40-69 years	56.62%	NA	2016	1997-2017	CTS②	5	③,④,⑥,⑦,⑧	Self-reported	NR	Self-reported	NOS	6

Soares	2017	Brazil	C	the 1993 Pelotas Birth Cohort	3576	Minors	51.05%	NA	Before Birth、18 years	18.4 years	Self-encoding	1	⑩	Self and mother report	NR	Trained personnel	NOS	8
Stannard	2022	UK	C	BCS70	7951	Minors	51.97%	NA	10 years	46 years	Self-encoding	1	⑩	Parental report	140/90	Trained personnel	NOS	8
Stein	2010	Americas, Europe, Asia	CS	WHM Surveys	18600	Adults	52.80%	21-98	NA	NA	Self-encoding	11	②,⑦,⑧,⑩,⑪,⑫,⑬,⑭,⑮,⑳,㉑,㉒	Face-to-face interviews	NR	Physician-confirmed Self-report	AHRQ	7
Sufen Chen	2022	China	CS	NA	4678	Minors	49.25%	8-19	NA	NA	ICAST-CH-C + CTQ-SF	5	②,⑥,⑦,⑧,⑫	Self-reported	WS/T610-2018	Trained personnel	AHRQ	9
Widom	2012	US	C	NA	1196	Minors	52.90%	NA	1967-1971	2003-2005	Official confirmation	3	①,②,⑦,⑧	Government records	140/90	Trained personnel	NOS	4
Wooldridge	2023	US	CS	NIAAA	36190	Adults	51.90%	46.2±0.2	NA	NA	Self-encoding	9	①,⑨,㉓	Self-reported	NR	Physician-confirmed Self-report	AHRQ	7

*ACE=adverse childhood experience; CS=cross-sectional; C=cohort; BP, blood pressure; BMI, body mass index;

*SES, socioeconomic status; CVD, cardiovascular disease; WMH, World Mental Health; NR, not reported;NA, not applicable.

*CFEQ, Childhood Family Environments Questionnaire； ACE-IQ， Adverse Childhood Experiences International Questionnaire;CTQ, Child Trauma Questionnaire;PIPES, Perceived Immigration Policy Effects Scale;CTQ-SF, a short form of the Childhood Trauma Questionnaire; SLC, the Survey of Living Conditions;CTS①, Conflict Tactics Scale; ETI, the Early Trauma Inventory; SCQ, self-completion questionnaire; SEP, social class and educational qualifications;EDS, the Everyday Discrimination Scale;FES-CV, Family Environment Scale-Chinese Version;RFQ, the Risky Families Questionnaire; FES, the Family Environment Scale; CTS②, Childhood Trauma Screener;ICAST-CH-C, ISPCAN child abuse screening tools-children's home Chinese version.

*CARDIA, the Coronary Artery Risk Development in Young Adults study； THIN， the Health Improvement Network;CHAMACOS, the Center for the Health Assessment of Mothers and Children of Salinas;MTC, the Mexican Teachers' Cohort;ALSPAC, the Avon Longitudinal Study of Parents and Children;NCS, the National Comorbidity Survey;FPSS, the Finnish Public Sector study; MECA, the Morehouse-Emory Cardiovascular; TILDA,the Irish Longitudinal Study on Ageing; MIDUS, the Mid-life in the United States;BRFSS, the Behavioral Risk Factor Surveillance System;NCDS： the National Child Development Study;CHARLS, China Health and Retirement Longitudinal Study;RICE, the Rape Impact Cohort Evaluation;SPMH, Sthe São Paulo Megacity Mental Health Survey;WMH, World Mental Health Surveys;Midwest Study, the Midwest Evaluation of the Adult Functioning of Former YFC;NHS

II, the Nurses' Health Study II;MUSP, the Mater Hospital-University of Queensland Study on Pregnancy;PCS3, in the Pittsburgh Common Cold Project 3; BCS70, the 1970 British Cohort Study;NIAAA, the National Epidemiologic Survey of Alcohol and Related Conditions-III;WS/T610-2018, Screening thresholds for high blood pressure in children and adolescents aged 7 to 18 years, published by the Chinese Health Commission in 2018.

*①abuse + neglect; ②neglect; ③emotional neglect; ④physical neglect; ⑤abuse; ⑥emotional abuse; ⑦physical abuse; ⑧sexual abuse; ⑨family dysfunction; ⑩parental divorce or separation; ⑪parental deaths; ⑫domestic violence; ⑬household mental illness; ⑭household substance abuse; ⑮household crime; ⑯peer bullying; ⑰community violence; ⑱group violence; ⑲one and more types of Ace; ⑳one or mild ACE;㉑two or moderate ACE; ㉒three ACE; ㉓four or severe Ace; ㉔accumulated per ACE.

Table S2. Association of different types and amounts of ACE with hypertension (total effect and geographic grouping)

ACE types	Hypertension				European				North America				Asian			
	N	OR	95%Conf.Interval		N	OR	95%Conf.Interval		Number	OR	95%Conf.Interval		N	OR	95%Conf.Interval	
Abuse + Neglect	4	1.14	1.01	1.29					3	1.07	0.94	1.22				
Neglect	9	1.20	1.12	1.29	2	1.21	1.10	1.34	3	1.40	1.11	1.76	1	1.09	0.92	1.30
Emotional neglect	3	1.07	1.05	1.09	2	1.07	1.05	1.09					1	3.44	1.27	9.32
Physical neglect	2	1.10	1.08	1.13	1	1.10	1.08	1.12								
Abuse	2	1.24	1.08	1.42					1	1.08	0.86	1.36	1	1.33	1.13	1.56
Emotional abuse	6	1.07	1.05	1.10	2	1.07	1.05	1.10	2	1.08	0.96	1.21	1	0.94	0.78	1.14
Physical abuse	10	1.09	1.07	1.11	2	1.08	1.06	1.10	4	1.24	1.15	1.34	1	0.89	0.75	1.05
Sexual abuse	11	1.05	1.02	1.08	2	1.02	0.99	1.05	5	1.31	1.21	1.42	1	0.97	0.78	1.20
Family dysfunction	3	1.09	1.06	1.13	1	1.07	1.03	1.11	2	1.24	1.13	1.37				
Parental divorce or separation	3	1.09	0.95	1.25	2	1.25	1.03	1.53								
Parental death	2	1.09	1.01	1.17					1	1.11	1.02	1.21				
Domestic violence	2	1.25	1.12	1.41												
Household mental illness	1	1.29	1.11	1.50												
Household substance abuse	1	1.30	1.08	1.57												
Household crime	1	1.33	1.09	1.62												
Community violence	1	1.53	1.15	2.04					1	1.53	1.15	2.04				
Group violence	2	1.09	0.74	1.61					2	1.09	0.74	1.61				
One and more types of ACE	5	1.08	1.04	1.12	1	1.42	1.26	1.60	4	1.05	1.01	1.09				
One or mild ACE	9	1.06	1.03	1.09					4	1.06	1.03	1.09	3	1.05	0.92	1.20
Two or moderate ACE	6	1.16	1.11	1.21					3	1.16	1.11	1.21	2	1.15	1.01	1.32
Three ACE	8	1.25	1.18	1.32					3	1.27	1.19	1.35	3	1.08	0.93	1.25
Four or severe ACE	7	1.46	1.36	1.56					3	1.55	1.42	1.69	4	1.34	1.20	1.49
Accumulated per ACE	4	1.06	1.05	1.07					2	1.08	1.06	1.11				

Table S3. Association of different types and amounts of ACE with hypertension (age subgroups)

ACE types	Minors				Middle-aged				Adults			
	N	OR	95%Conf.Interval		N	OR	95%Conf.Interval		N	OR	95%Conf.Interval	
Abuse + Neglect	1	1.07	0.74	1.54	**1**	**1.62**	**1.18**	**2.23**	1	1.07	0.93	1.24
Neglect	**3**	**1.16**	**1.05**	**1.28**	**2**	**1.42**	**1.11**	**1.81**	**3**	**1.21**	**1.03**	**1.41**
Emotional neglect					**1**	**3.44**	**1.27**	**9.32**				
Physical neglect												
Abuse	**1**	**1.33**	**1.13**	**1.56**					1	1.08	0.86	1.36
Emotional abuse	3	1.02	0.92	1.13	**1**	**1.57**	**1.16**	**2.13**	1	1.07	0.91	1.25
Physical abuse	4	0.96	0.85	1.08	**3**	**1.27**	**1.18**	**1.38**	**2**	**1.38**	**1.20**	**1.59**
Sexual abuse	4	0.95	0.81	1.10	**3**	**1.49**	**1.35**	**1.63**	3	0.96	0.82	1.14
Family dysfunction									**3**	**1.09**	**1.06**	**1.13**
Parental divorce or separation	1	1.19	0.93	1.51					2	1.05	0.89	1.24
Parental death					**1**	**1.11**	**1.02**	**1.21**	1	1.02	0.89	1.17
Domestic violence									**2**	**1.25**	**1.12**	**1.41**
Household mental illness									**1**	**1.29**	**1.11**	**1.50**
Household substance abuse									**1**	**1.30**	**1.08**	**1.57**
Household crime									**1**	**1.33**	**1.09**	**1.62**
Community violence	**1**	**1.53**	**1.15**	**2.04**								
Group violence	1	1.10	0.43	2.82	1	1.09	0.71	1.67				
One and more types of ACE					**1**	**1.13**	**1.06**	**1.21**	**5**	**1.05**	**1.02**	**1.08**
One or mild ACE					3	1.04	0.99	1.08	**5**	**1.09**	**1.04**	**1.14**
Two or moderate ACE					**2**	**1.12**	**1.05**	**1.20**	**3**	**1.19**	**1.13**	**1.25**
Three ACE					**2**	**1.37**	**1.24**	**1.52**	**4**	**1.24**	**1.16**	**1.34**
Four or severe ACE	**1**	**5.72**	**3.27**	**10.01**	**1**	**1.59**	**1.42**	**1.78**	**3**	**1.62**	**1.46**	**1.80**
Accumulated per ACE					**1**	**1.13**	**1.05**	**1.21**	**3**	**1.06**	**1.05**	**1.07**
